# Clinical Outcome in Patients with Large Sinonasal Tumors with Intracranial Extension

**DOI:** 10.1055/a-2082-4951

**Published:** 2023-05-24

**Authors:** Max E. Keizer, Koos E. Hovinga, Martin Lacko, Danielle B.P. Eekers, Laura W.J. Baijens, Bernd Kremer, Yasin Temel

**Affiliations:** 1 Department of Neurosurgery, Maastricht University Medical Centre + , Maastricht, The Netherlands; 2 Dutch Academic Alliance Skull Base Pathology, Maastricht/Nijmegen, The Netherlands; 3 Department of Otorhinolaryngology, Head and Neck Surgery, Maastricht University Medical Centre + , Maastricht, The Netherlands; 4 School for Oncology and Reproduction (GROW), Maastricht University Medical Centre + , Maastricht, The Netherlands; 5 Department of Radiation Oncology (Maastro), Maastricht, The Netherlands

**Keywords:** Skull base, esthesioneuroblastoma, sinonasal carcinoma, inverted papilloma, squamous cell carcinoma, adenoid cystic carcinoma, radiotherapy, reconstruction, craniotomy

## Abstract

**Objectives** Malignant tumors of the sinonasal cavities with extension to the frontal skull base are rare and challenging pathologies. Combined-approach surgery using a frontobasal craniotomy and endoscopic sinus surgery with reconstruction of the anterior skull base followed by adjuvant radiotherapy is a preferred treatment strategy in selected cases. Morbidity and mortality rates are high in this population. We aim to add our experience to the current literature.

**Design** We performed a retrospective cross-sectional single center study of the long-term clinical outcome in a tertiary university referral hospital in the Netherlands between 2010 and 2021. Descriptive statistics and frequency distributions were performed

**Participants** Patient, tumor, treatment, complications and survival characteristics of eighteen consecutive patients were extracted from the electronic health records.

**Main Outcome Measures** The primary outcome measures are progression free survival, overall survival and complication rate.

**Results** Eighteen consecutive patients were included with a mean age of 61 (SD ± 10) years (range 38-80); ten males and eight females. Gross total resection was achieved in 14 (77%) patients. Eleven (61%) patients underwent local radiotherapy, one (5%) chemotherapy and three (17%) a combination of both. Mean follow-up duration was 49 months (range 3 – 138). Three (17%) patients died in hospital due to post-operative complications. Six (33%) patients died during follow-up due to disease progression. Mean progression-free survival was 47 months (range 0 – 113).

**Conclusion** In conclusion, the overall survival was 50% for this group of patients with large sinonasal tumors. Progressive disease affects survival rate severely. Surgical complications were seen in five (28%) patients. Radiotherapy is associated with high complication rates. Radiation necrosis was a serious complication in two patients and could be treated with high dose steroids.

## Introduction

Sinonasal tumors are rare and challenging pathologies, accounting for less than 1% of all malignancies. 1 These lesions often present in an advanced stage, involving both paranasal sinuses and surrounding structures such as the anterior skull base, brain, and orbit. 1 When the skull base is affected, the tumor may have invaded the dura and brain parenchyma worsening the prognosis. 2 The treatment modality of these lesions depends on the extent of the lesion and histopathological diagnosis. 2 3 Open approaches used to be the only surgical option; however, in the last three decades, endoscopic approaches are added as part of the surgical strategy. Current literature suggests a reduced complication rate in endoscopic approaches compared with open surgery while preserving comparable oncologic outcomes. The choice for specific surgical strategies also depends on preferences and experiences of the surgical team. Extensive invading tumors might be less suitable for endoscopic surgery alone. In tumors eligible for resection, surgical removal followed by skull base reconstruction is the first step. 4 5 6 Depending on the type of tumor and disease stage, postoperative chemo and/or radiation therapy is indicated. 7 8 9 10 11 12 13 14 15

The most common tumor is squamous cell carcinoma (SCC), accounting for up to 50% of all sinonasal malignancies, followed by adenocarcinoma (AC, 12.6%), melanoma (ML, 6.6%), esthesioneuroblastoma (ENB, 6.3%), adenoid cystic carcinoma (ACC, 6.2%), and sinonasal undifferentiated carcinoma (SNUC, 3.1%). 1 Most patients are diagnosed at an advanced stage where the cancer has invaded surrounding structures (54.5%) or show distant metastasis (14.9%). 16 17 A distinct minority presents in an early, local disease stage (30.6%). 1 Overall survival is poor with an average 5-years survival of 54.5%. 1 However, subgroups of survival rates may be stratified according to histology and disease stage. 16 17 A relatively good prognosis is seen in ENB and ACC with a 5-years overall survival of 71.0 and 69.5%, respectively, and in patients with only local disease (80.9%). Intermediate survival rates at 5 years are seen in SCC (53.0%) and AC (63.0%) and in patients with regionally advanced disease (48.6%). The poorest prognosis is observed in patients with ML and SNUC with only 34.7% survival after 5 years and patients with distant metastasis (28.2%). 1

Surgical resection is the mainstay treatment for these tumors. Gross total resection is associated with a better outcome compared with subtotal removal and with radiotherapy mono-treatment. 7 Radiotherapy can be applied as part of palliative treatment and as adjuvant therapy. 7 Surgery for tumor recurrence may be considered in selected cases but is associated with high morbidity and mortality. 2 The benefit of induction or adjuvant chemoradiation is still unclear. 15

Treatment of sinonasal cancer is associated with high rates of complications, both surgery and radiotherapy related. Postoperative mortality is around 4.7% and comorbidity is associated with higher surgery-related mortality. 18 Complications are seen in 36.3% of the patients. 18 Wound-related complications (infection, dehiscence, and necrosis) occur in 19.8%, central nervous system (CNS)-related complications (cerebral spinal fluid leak, meningitis, pneumocephalus, and neurological deficit) in 16.2%, systemic complications (cardiovascular, pulmonary, and urinary tract infection) in 4.8%, and orbital complications (blindness, diplopia, and lacrimal duct obstruction) in 1.7%. The presence of medical comorbidity, induction radiotherapy, and dural or brain invasion is associated with higher probability of complications. 18

Radiotherapy-associated complications are reported in up to 50% of patients. These include all five severity grades in accordance with the Common Terminology Criteria for Adverse Events. 19 Most common complications are visual deficits up to blindness, tear film imbalance, nasal mucosa inflammation, wound infection, bone flap necrosis, brain necrosis, pituitary deficit, and pain. 11 12 13 14 18

The low incidence of these tumors and their heterogeneous aspect makes it difficult to execute large randomized controlled trials. Therefore, mostly papers reporting on small retrospective cohorts are available. We would like to add to the current literature by describing our single institute long-term outcome on the treatment of large sinonasal cancers with intracranial extension. The aim of this study is to evaluate tumor control, progression free and overall survival, and therapy-associated complications after a frontobasal craniotomy with endoscopic assistance and skull base reconstruction.

## Materials and Methods

### Study Design

We conducted a retrospective analysis of our cohort between January 2010 and December 2021. Data including medical history, neurological status, radiological examinations, operative details, surgical outcome, histopathology, adjuvant therapies, complications, and follow-up details were extracted from the electronic patient files. Tumor recurrence and clinical outcome data were collected at 3 and 12 months postoperative time-points and at the last follow-up time-point. The study protocol was approved by the medical ethics committee of Maastricht University Medical Centre+ and received the following reference number: 2021-2608.

### Subjects

All consecutive patients with a sinonasal tumor with anterior skull base extension treated using a bifrontal (frontobasal) craniotomy by our multidisciplinary skull base team were eligible to be included in this study. Case-by-case discussions took place, and the best possible tailored therapy was proposed to the patient. Patients had to have a Karnofsky performance score above 60 to be eligible for resection and a gross total resection has to be possible. In our institution, a bifrontal craniotomy is used in all cases with stage T4b carcinoma. Stage 4a carcinoma can be treated by an endoscopic approach or a bifrontal craniotomy. The approach is selected based on anatomical location and whether the surgeon believes the local invasion can be removed and reconstructed by endoscopy, if not, a bifrontal craniotomy is indicated. The follow-up was performed by the multidisciplinary skull base team.

### Surgical Approach

The approach in all cases was the transcranial route (here referred as frontobasal craniotomy) to the paranasal sinuses and removal of tumor tissue along this route. Endoscopy was used through the transcranial opening and/or transnasal to assist tumor removal. The details of the craniotomy were as follows. A bifrontal incision was performed with preservation of the periosteum to be used for the skull base reconstruction. When a second surgery was necessary, then the galea aponeurotica layer was used for skull base reconstruction. After the bifrontal craniotomy, the dura was opened if there was an intradural tumor. For the intradural approach, the most anterior part of the superior sagittal sinus was ligated and the falx cerebri was transected. The following step was to remove the posterior wall of the frontal sinus, drilling of the cribiform plate and drilling of the cranial part of the sphenoid plane to access the frontal, ethmoidal, and sphenoid sinuses, respectively. Tumor tissue was removed using microsurgical techniques. The CUSA-aspirator was applied as well as the surgery shaver, whenever applicable. When the intraorbital viscera were involved in the tumor process, an exenteration was performed and the craniofacial surgeon prepared the orbit for an ocular prosthetic. When gross total resection was achieved, reconstruction of the anterior skull base was performed. A split skull graft from the frontal craniotomy bone was made and inserted at the frontal skull base opening, and then the periosteum was used as cover supported by a fat graft from the para umbilical region. The wound was closed in a layered fashion. All patients received 7 days antibiotic prophylaxis postoperatively and 2 days of bedrest.

### Adjuvant Therapy

In our institution, all patients with a T4 sinonasal carcinoma receive adjuvant local regional radiotherapy. In line with this, the patients in this series received photon radiation therapy, consisting of 30 to 35 fractions of 2.0 Gy to a total dose of 60 to 70 Gy using volumetric modulated arc therapy. The target volume consisted of the resection cavity and tumor remnant after resection with a margin according to local guidelines. Timing depended on wound healing and complications and varied between 2 to 6 weeks postoperatively.

Induction chemotherapy is not applied in our institution. Adjuvant systemic therapy is an option in patients with recurrent disease who are not eligible for reresection or with distant metastases.

### Clinical Follow-up

**Fig. 1:**
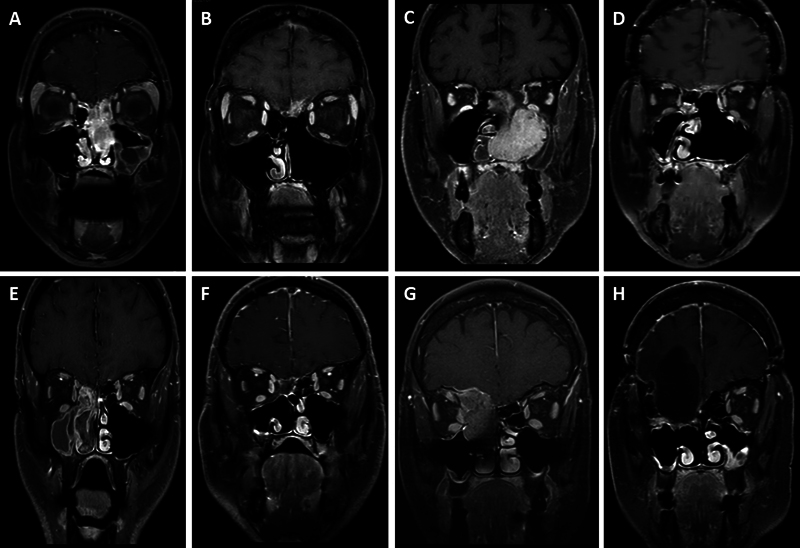
Radiological imaging of sinonasal cancers. This figure demonstrates four cases with a pre- and postoperative contrast enhanced T1 MRI in the coronal plane. ( **A** and **B** ) Case 4 with an esthesioneuroblastoma. ( **C** and **D** ) Case 2 with an adenoid cystic carcinoma. ( **E** and **F** ) Case 7 with an adenocarcinoma. ( **G** and **H** ) Case 8 with a squamous cell carcinoma, an orbital exenteration has been performed to achieve gross total resection.

Oncological follow-up was performed at 3 and 6 months postoperatively after which the standard interval remained 6 months. A standardized examination protocol, used in daily clinical practice was performed. This protocol comprised a physical examination with endoscopic evaluation of the nasal cavity and opened paranasal sinuses. A magnetic resonance imaging (MRI) was performed to evaluate tumor recurrence. See Fig. 1 for an example. All cases that showed progressive disease or symptoms were discussed in our skull base board.

### Statistics

A descriptive approach was used to display the data due to the small and heterogeneous nature of our cohort. All data were analyzed using Statistical Package for the Social Sciences version 27 software (SPSS, Chicago, IL, United States).

## Results

### Demographics

In total 18 patients were identified for inclusion, 10 males and 8 females. Mean age at surgery was 61 years (range: 38–80). Three (16%) patients were diagnosed with an ENB, three (16%) with an adenoid cystic sinonasal carcinoma, five (28%) with an AC, two (11%) with an SCC, two with an SNUC, one (5%) with an ML, one with an inverted papilloma with SCC differentiation and one patient with a sinonasal neuroendocrine carcinoma. Most patients presented with complaints of nasal obstruction ( *n* = 10) and rhinorrhea ( *n* = 8). Two patients suffered from severe frontal lobe syndrome due to significant cytotoxic edema and mass effect on the frontal lobes. One patient had an epileptic seizure as her presenting symptom. Twelve patients had orbital involvement of the tumor process, six of which showed symptoms following this involvement. See Table 1 for a more detailed description.

**Table 1:** Demographics

Case number	Sex	Age (year)	Presenting symptoms	Histopathology	Oncological staging	Radiological extension
1	F	74	Nasal obstruction, rhinorrhea	Esthesioneuroblastoma	cT4bN0M0	Nasal sinus, orbit
2	F	69	Nasal obstruction, rhinorrhea	Adenoid cystic carcinoma	cT4bN0M0	Nasal sinus, orbit, anterior fossa
3	M	59	Nasal obstruction, rhinorrhea	Inverted papilloma with squamous cell carcinoma	cT4bN0M0	Nasal sinus, orbit, anterior fossa
4	F	38	Nasal obstruction, rhinorrhea	Esthesioneuroblastoma	cT4bN0M0	Nasal sinus, anterior fossa
5	F	61	Epilepsy	Adenoid cystic carcinoma	cT4bN0M0	Nasal sinus, orbit, anterior fossa
6	M	69	Nasal obstruction, rhinorrhea, anosmia	Esthesioneuroblastoma	cT4bN0M0	Nasal sinus, anterior fossa
7	M	55	Nasal obstruction, rhinorrhea, epistaxis	Adenocarcinoma	cT4bN0M0	Nasal sinus, anterior fossa
8	F	60	Visual deficit and diplopia	Squamous cell carcinoma	cT4bN0M0	Nasal sinus, orbit, anterior fossa
9	F	80	Nasal obstruction, epistaxis	Sinonasal neuro-endocrine carcinoma	cT4bN0M0	Nasal sinus, orbit, anterior fossa
10	F	73	Nasal obstruction, rhinorrhea, epistaxis	Melanoma	cT4aN0M0	Nasal sinus, orbit, anterior fossa
11	M	66	Frontal lobe syndrome	Adenocarcinoma	cT4bN0M0	Nasal sinus, anterior fossa
12	M	58	Nasal obstruction, diplopia, dacryorrhea	Squamous cell carcinoma	cT4aN0M0	Nasal sinus, orbit, anterior fossa
13	M	48	Nasal obstruction, epistaxis	Adenocarcinoma	cT4bN0M0	Nasal sinus, orbit, anterior fossa
14	M	56	Frontal lobe syndrome	Adenocarcinoma	cT4aN0M0	Nasal sinus, anterior fossa
15	M	64	Headache, anosmia, visual field deficit	Sinonasal undifferentiated carcinoma	cT4bN0M0	Nasal sinus, orbit, anterior fossa
16	F	50	Visual deficit, diplopia, anosmia	Adenoid cystic carcinoma	cT4bN0M0	Nasal sinus, orbit, anterior fossa
17	M	66	Epistaxis	Adenocarcinoma	cT4bN0M0	Nasal sinus, anterior fossa
18	M	52	Rhinorrhea, anosmia, diplopia	Sinonasal undifferentiated carcinoma	cT4bN0M0	Nasal sinus, orbit, anterior fossa

Abbreviations: F, female; M, male.

Note: This table shows demographic data consisting of case nr, sex, age at surgery, presenting symptoms at diagnosis, the histological diagnosis, oncological staging and the extension of the tumor borders.

### Clinical Course and Survival

The mean length of stay in the hospital was 21 days (range: 4–132). Gross total resection was achieved in 14 (77%) patients. Eleven patients (61%) had no complications during their hospital stay. Three (17%) patients died in the hospital due to postoperative complications consisting of subarachnoid hemorrhage (SAH), cardiac arrest, and ventriculitis, respectively.

The mean follow-up duration was 49 months (range: 3–138). Six (33%) patients died during follow-up. Mean progression-free survival was 47 months (range: 0–113).

The individual cases will be discussed per tumor type. See Table 2 for a more detailed description.

**Table 2:** Clinical course and survival stratified toward tumor type

Case number	Histopathology	Hospital length of stay (days)	Complications in hospital	Progression-free survival (months)	Total survival (months)
1	Esthesioneuroblastoma	11	No	20	20
4	Esthesioneuroblastoma	10	No	15	51
6	Esthesioneuroblastoma	8	No	64	64 a
2	Adenoid cystic carcinoma	9	No	66	66
5	Adenoid cystic carcinoma	–	–	100	138
16	Adenoid cystic carcinoma	17	No	6	18
7	Adenocarcinoma	7	No	58	58
11	Adenocarcinoma	22	Liquorrhea	78	78
13	Adenocarcinoma	13	No	6	13 a
14	Adenocarcinoma	26	Delirium	6	7 a
17	Adenocarcinoma	4	SAH, HCP	–	– a
8	Squamous cell carcinoma	15	No	113	113
12	Squamous cell carcinoma	8	No	0	4 a
15	Sinonasal undifferentiated carcinoma	9	No	7	8 a
18	Sinonasal undifferentiated carcinoma	21	Liquorrhea, meningitis, pulmonary embolism	–	3 a
3	Inverted papilloma with squamous cell carcinoma	136	Hydrocephalus, ventriculitis, pneumonia,	–	– a
9	Sinonasal neuro-endocrine carcinoma	28	No	94	94
10	Melanoma	13	Liquorrhea, cardiac arrest	–	– a

Abbreviations: HCP, hydrocephalus; SAH, subarachnoid hemorrhage.

Note: Cases are categorized in accordance to their histopathology; hospital stay is presented in days, progression free and overall survival is presented in months.

a deceased.

#### Adenocarcinoma

Case 7 had no complications postoperatively and is now 58 months after surgery without signs of tumor recurrence. Case 11 showed at postoperative day two nasal liquorrhea, which was resolved with an external lumbar drain and acetazolamide for 7 days. This patient is now 78 months in follow-up without tumor recurrence. Case 13 had no in-hospital complications, however, showed distant cutaneous and osseous tumor recurrence after 6 months for which systemic therapy and locoregional radiotherapy were given. After 13 months, the patient died due to progressive disease. Case 14 experienced a delirium postoperatively, probably related to the preoperative already present bifrontal edema. It resolved using antipsychotic medication. Six months postoperatively, the patient had distant leptomeningeal tumor recurrence. The patient refused chemoradiation and died shortly after. Case 17 had a severe intra-operative complication, an SAH, from which the patient died 3 days postoperatively. More details are given in the next section.

#### Esthesioneuroblastoma

None of the patients diagnosed with an ENB suffered from in-hospital complications. Case 1 is 20 months in follow-up in which there is no evidence for tumor recurrence.

Case 4 refused postoperative radiotherapy and showed tumor recurrence at 15 months after surgery. After discussions with patient and parents, consent was given for reresection with postoperative radiotherapy. The patient is now 51 months in follow-up with no sign of tumor recurrence.

Case 6 has died 64 months after surgery due to the late complications effects of surgery and radiotherapy. The patient suffered from epilepsy and a frontal lobe syndrome with bifrontal radio necrosis and infection. Patient's condition deteriorated fast, without signs of tumor recurrence, and died

#### Adenoid Cystic Carcinoma

One case had an incomplete patient record. The other two cases had an uncomplicated hospital stay. Case 2 is now 66 months in follow-up without signs of tumor recurrence.

Case 5 has data available since 2012. The patient showed tumor recurrence 100 months postoperative for which reresection with adjuvant radiotherapy was advised; however, patient refused treatment. The patient was referred to an external oncologist for systemic therapy. Currently, the patient has shown minimal progressive disease after 138 months postoperative. Case 16 showed tumor recurrence at 6 months postoperatively for which the patient was referred to an external oncologist for systemic therapy. The patient is now in remission at 18 months follow-up.

#### Squamous Cell Carcinoma

Case 8 underwent an uncomplicated hospital stay and is now 113 months in follow-up without tumor recurrence. Case 12 had no complications in the first clinical period; however, only a subtotal resection was achieved; therefore, a second operation was needed to achieve curation. However, 4 months after the first operation patient showed pulmonary metastasis and refused further treatment. Patient died shortly after.

#### Sinonasal Undifferentiated Carcinoma

Case 15 had no in-hospital complications. After 7 months of follow-up, the patient showed loco-regional recurrence for which no curative treatments were available. The best supportive care protocol was applied and the patient died shortly after.

Case 18 had a major complicated clinical stay, for more details see the next section. The patient showed rapid disease progression after locoregional and systemic treatment. The best supportive care protocol was started, and patient died shortly after.

#### Miscellaneous

Case 3 had a severely complicated hospital stay and died after more than 4 months of treatment, for more details see the next section.

Case 9 had an uncomplicated hospital stay and is now 94 months in follow-up without tumor recurrence.

Case 10 died from an in-hospital cardiac arrest, for more details see the next section.

### Complications of Surgery

We recorded surgical complications in 5 out of 18 patients (28%). Three (17%) patients died during the direct postoperative period. Five (28%) patients needed a reoperation due to a complication. See Table 3 for a more detailed description of each individual case.

**Table 3:** Details on surgery stratified toward tumor type

Case number	Histopathology	Procedure	Extent of resection	Complication	Reoperation
1	Esthesioneuroblastoma	Bifrontal + endoscopy	Gross total	Late wound infection after RTx	Wound revision with bone flap removal
4	Esthesioneuroblastoma	Bifrontal + endoscopy	Gross total	–	Re-resection regrowth
6	Esthesioneuroblastoma	Bifrontal + endoscopy	Gross total	Late wound infection after RTx, pneumencephaly, epilepsy	Wound revision with bone flap removal and resection radio necrosis
2	Adenoid cystic carcinoma	Bifrontal + endoscopy	Gross total	–	–
5	Adenoid cystic carcinoma	Bifrontal	Gross total	Late wound infection after RTx, pneumencephaly	Wound revision with bone flap removal
16	Adenoid cystic carcinoma	Bifrontal + endoscopy	Subtotal	–	–
7	Adenocarcinoma	Bifrontal + endoscopy	Gross total	–	–
11	Adenocarcinoma	Bifrontal	Gross total	Liquorrhea	External lumbar drainage 7 d + acetazolamide
13	Adenocarcinoma	Bifrontal + endoscopy	Gross total	Late wound infection after RTx	Wound revision with bone flap removal
14	Adenocarcinoma	Bifrontal + endoscopy	Gross total	–	–
17	Adenocarcinoma	Bifrontal	Partial	SAH, hydrocephalus	External lumbar drain, external ventricular drain, craniectomy
8	Squamous cell carcinoma	Bifrontal + endoscopy + exenteration	Gross total	Late orbital liquorrhea after RTx	External lumbar drain and combined reconstruction orbit
12	Squamous cell carcinoma	Bifrontal + endoscopy	Subtotal	–	Orbit exenteration and lat rhinotomy for remnant tumor
15	Sinonasal undifferentiated carcinoma	Bifrontal + endoscopy	Gross total	–	–
18	Sinonasal undifferentiated carcinoma	Bifrontal + endoscopy	Subtotal	Nasal liquorrhea, meningitis,	External lumbar drain
3	Inverted papilloma with squamous cell carcinoma	Bifrontal + endoscopy	Gross total	Hydrocephalus, ventriculitis, cerebral edema	Multiple external and internal shunt revisions
9	Sinonasal neuro-endocrine carcinoma	Bifrontal + endoscopy	Gross total	–	–
10	Melanoma	Bifrontal + lat rhinotomy + exenteration	Gross total	Liquorrhea, cardiac arrest	External lumbar drain for 5 d

Abbreviations: RTx, radiotherapy; SAH, subarachnoid hemorrhage.

Note: Cases are organized according to tumor type. We report of the operation strategy; bifrontal craniotomy, lateral rhinotomy, endoscopic assistance and orbital exenteration. Additionally, we provide the degree of resection; gross total >90%, subtotal 80 to 90% and partial <80%. Finally, the treatment related complication is described and the concurrent treatment.

#### Wound Infection

No patient suffered from direct postoperative wound infection. However, four patients got late postradiotherapy wound problems requiring operative intervention.

Cases 1, 5, and 13: postradiation wound infection requiring wound revision and removal of the bone flap, 4, 72, and 6 months, respectively. Case 6 also underwent wound revision for a chronic infection, in addition, a partial resection of the frontal lobe was performed to get histopathological samples excluding tumor recurrence and proving radio necrosis.

#### Liquorrhea

Three patients (17%) suffered from direct postoperative liquorrhea, all were treated successfully using an external lumbar drain, one with an addition of acetazolamide. We report one late orbital liquorrhea following radiotherapy and a chronic infection requiring reconstructive surgery.

Case 11: nasal liquorrhea requiring 7 days of external lumbar drainage and acetazolamide

Case 8: uncomplicated clinical period. Eight months postoperatively orbital liquorrhea with frontal lobe herniation and chronic infection. First, an external lumbar drain was placed; this did not resolve the liquorrhea. Therefore, a combined approach with the plastic surgeon was used to reconstruct the orbital region of the frontal skull base using a temporalis fascia and split skin graft. Postoperative wound healing was uneventful.

Case 18: postoperative nasal liquorrhea and subsequent neurological deterioration based on pseudomonas meningitis occurred. Treatment with an external lumbar drain and antibiotics was given. In the intensive care unit, patient showed respiratory worsening based on pneumonia and pulmonary embolism which were treated using antibiotics and low-molecular weight heparin in therapeutic dosages. The patient recovered and was discharged from the hospital on day 21 postoperatively.

Case 10: postoperative nasal liquorrhea occurred which was treated with an external lumbar drain. On postoperative day 13 patient had a cardiac arrest, with unknown cause and unfortunately unsuccessful resuscitation. The family gave no permission for postmortem analysis.

#### Intra-operative Cerebral Swelling

**Fig. 2:**
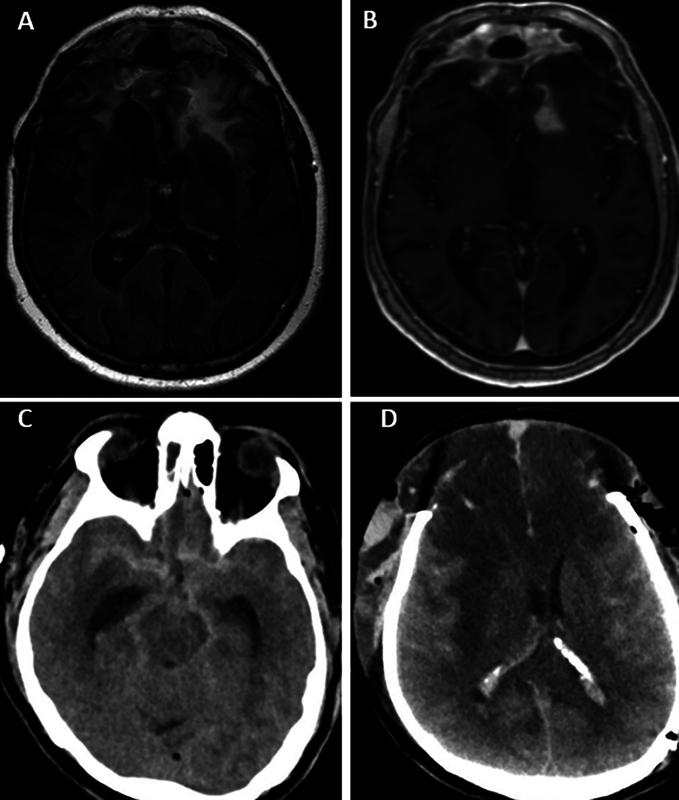
Radiological imaging of complications. This figure demonstrates two cases with complications. ( **A** and **B** ) Case 6 with bifrontal radio necrosis, A is a FLAIR T2 weighted axial image showing bifrontal hyperintense signal corresponding with subcortical edema. B is a T1 + gadolinium axial image demonstrating intraparenchymal and subcutaneous contrast enhancement suggestive of radio necrosis and/or infection. ( **C** and **D** ) Case 17 with per-operative subarachnoid hemorrhage and cerebral swelling. C is a CT axial image direct post-operative demonstrating diffuse SAH in the basal cisterns and both sylvian fissures with enlarged temporal horns and diffuse swelling. D is a CT axial image after bone flap removal and occipital ventricular catheter placement. The image demonstrates large hypo dense, ischemic, areas bifrontal, herniating frontal lobes, and diffuse swelling.

Case 17: Intraoperative severe cerebral swelling occurred necessitating the premature ending of the procedure. Postoperative CT-scan demonstrated an SAH with intraventricular expansion (fisher grade 4) without evidence of an aneurysm on the CT-angiogram. An external lumbar drain was placed to treat hydrocephalus. The clinical status deteriorated, and the lumbar drain was replaced by an external occipital catheter and removal of the bifrontal boneflap. Follow-up CT scan showed further expansion of the SAH and an additional ICH in the right frontal lobe, see Fig. 2 . Patient had a Glasgow Coma Score of E1M1Vtube without brain stem reflexes. No additional therapies were available and patient died shortly after extubation. There was no approval for postmortem analysis.

#### Postinfection Hydrocephalus and Refractory Ventriculitis

Case 3: Patient presented with a severe frontal lobe syndrome based on a right frontal intraparenchymal streptococcus anginosus abscess and a sinonasal carcinoma. The abscess was aspirated and antibiotics were started. Patient improved rapidly, however, follow-up MRI demonstrated growth of the abscess, possibly due to a defect of the anterior skull base. In the skull base board, it was decided to perform a combined approach to achieve maximal tumor and abscess resection and close the skull base defect. Postoperatively patient did not regain normal consciousness due to massive bifrontal edema for which a high dosage of dexamethasone and mannitol was administered. The patient slowly improved, however, a second deterioration based on a hydrocephalus and ventriculitis led to the placement of an external ventricular catheter. After over 3 months of treatment, including multiple drain revisions, a refractory ventriculitis was concluded. In combination with an ongoing oncological process, further medical treatment was considered obsolete. Best supportive care protocol was started, and patient died 4 days later.

### Complications of Radiation

Of 14 patients receiving postoperative radiotherapy, 7 (50%) had a complication linked to the radiotherapy. We report on each type of complication below. See Table 4 for a more detailed description.

**Table 4:** Radiotherapy complications according CTCAE version 5

Case number	Histopathology	Prescribed radiation dose	Complications	Grade and timing
1	Esthesioneuroblastoma	33 × 2 Gy	Wound dehiscence and infection, tear film instability	4, late; 3, early
4	Esthesioneuroblastoma	Post re-resection 33 × 2 Gy	–	
6	Esthesioneuroblastoma	33 × 2 Gy	Radio necrosis, frontal lobe syndrome, epilepsy, pituitary deficit	4, late
2	Adenoid cystic carcinoma	33 × 2 Gy	–	
5	Adenoid cystic carcinoma	33 × 2 Gy	Radio necrosis, nasal liquorrhea, wound infection	4, late
16	Adenoid cystic carcinoma	35 × 2 Gy	–	
7	Adenocarcinoma	33 × 2 Gy	–	
11	Adenocarcinoma	33 × 2 Gy	Radio necrosis, pituitary deficit	4, late
13	Adenocarcinoma	33 × 2 Gy	Wound infection	4, early
14	Adenocarcinoma	33 × 2 Gy	–	
17	Adenocarcinoma	–	–	
8	Squamous cell carcinoma	33 × 2 Gy	Nasal synechiae, radio necrosis	4, late
12	Squamous cell carcinoma	35 × 2 Gy	Wound dehiscence orbit	4, early
15	Sinonasal undifferentiated carcinoma	35 × 2 Gy	–	
18	Sinonasal undifferentiated carcinoma	–	–	
3	Inverted papilloma with squamous cell carcinoma	–	–	
9	Sinonasal neuro-endocrine carcinoma	33 × 2 Gy	–	
10	Melanoma	–	–	

Abbreviation: CTCAE, Common Terminology Criteria for Adverse Events.

Note: Cases are organized according to tumor type. We report on the radiotherapy scheme; the first number is the amount of fractions, the second is the fractionated dosage in gray. Finally, we describe the radiotherapy related complications and their severity grade according to the current guidelines. 19 We differentiate between early and late occurrence.

#### Wound Infection

We observed five late wound infections (28%), in a range of 1 week to 65 months after radiotherapy, four of which needed a reoperation.

Case 1: 4 months postoperative and 1 week after finishing radiotherapy patient reported subcutaneous swelling and wound dehiscence. The wound was revised, and the bone flap was removed after which the recovery was completed uneventful.

Case 6: 33 months postradiotherapy, the patient underwent reoperation for wound dehiscence and biopsy of suspected tumor recurrence/radio necrosis, see Fig. 2 . Analysis demonstrated no residual tumor.

Case 5: 65 months postradiotherapy the patient got nasal liquorrhea and pneumencephaly, which recovered with a conservative approach. 72 months postradiotherapy the patient got ocular pain and wound dehiscence based on a bone flap infection for which the wound was revised and the bone flap removed. Postoperative period was uneventful.

Case 13: 1 week postradiotherapy wound infection. Three times antibiotic regime improved clinical status; however, 6 months postradiotherapy persistent wound infection, the wound was revised and the bone flap was removed. This case had an uneventful postoperative recovery.

Case 12: 1 week after start radiotherapy a wound infection of the orbit developed requiring antibiotics and recurrent nasal flushing, which led to full recovery.

#### Ocular Symptoms

Case 6: The ophthalmologist diagnosed instable tear film and asymmetrical cataract 6 months postradiotherapy which was treated using supplementary tears.

#### Pituitary Gland Deficits

Case 6: 50 months postradiotherapy patient was diagnosed with pituitary gland deficits needing supplementation.

Case 11: 36 months postradiotherapy diagnosed with pituitary gland deficits requiring supplementation.

#### Radio Necrosis

We observed four (22%) patients with radiological radio necrosis of the brain parenchyma; two were symptomatic (11%) requiring dexamethasone.

Case 6: 9 months postradiotherapy patient had an epileptic seizure, presumably resulting from bifrontal radio necrosis, see Fig. 2 . Sixteen months postoperatively patient developed a severe frontal lobe syndrome due to bifrontal edema, which was treated with dexamethasone for 3 months and cognitive rehabilitation. The patient remained care dependent.

Case 8: 8 months postoperative frontal lobe radio necrosis requiring dexamethasone for 3 months and cognitive rehabilitation. The patient remained care dependent.

Case 5: 24 months postradiotherapy asymptomatic frontal radio necrosis, which required no treatment.

Case 11: 24 months postradiotherapy asymptomatic frontal radio necrosis, which required no treatment.

#### Nasal Symptoms

Case 8: 6 months postradiation severe nasal synechiae with substantial nasal obstruction needing cleaving.

## Discussion

Sinonasal cancers are rare and challenging pathologies. 17 Often, they present in an advanced stage, making a gross total resection less probable. 17 Moreover, this group of tumors is histologically very heterogeneous making comparisons difficult and interpretations more general. Additionally, the current treatment paradigm, which consists of surgical radical resection, possibly followed by adjuvant chemo and/or radiation, is associated with high rates of morbidity and mortality. 15 16 17 18 In our retrospective cohort study, we report on our own experience of 18 cases with sinonasal cancers with anterior fossa destruction treated by an endoscope-assisted frontobasal craniotomy followed by autologous skull base reconstruction. The cohort consists of eight different histological diagnoses. We achieved gross total resection in 14 patients (78%). Radiotherapy was given postoperatively to 14 patients, three of which were combined with chemotherapy. Half of the patients died; three patients died during the immediate postoperative period and six patients died during a mean follow-up of 47 months. Five of those six patients showed disease progression within 7 months. We recorded surgical complications in five patients and radiotherapy-related complications in seven patients.

### Progression-Free and Overall Survival

Our cohort showed a mean progression-free survival of 47 months. Overall survival was 50% at a mean follow-up of 49 months. These results are in line with reports in the literature. 7 8 16 17 20 21 The most important predictor of survival is achieving gross total resection while preserving quality of life. 22 We achieved complete resection in 14/18 patients, one partial resection was due to a intra-operative complication. The other three patients with a subtotal tumor resection showed early recurrence between 3 and 6 months; one was treated with reresection and systemic chemotherapy; however, the patient remained progressive. The other was given systemic chemotherapy and is in remission at 18 months. The third patient had a major complicated disease course and was progressive under chemo-radiation.

Multimodality management with a combination of surgery and chemoradiation is the preferred management strategy. 3 15 16 17 The absence of large randomized controlled trials makes the evidence of low quality; however, in selected cohorts, a multimodal treatment gives greater locoregional tumor control and longer survival rates. 23 24 25 All patients eligible for adjuvant radiotherapy received fractionated loco-regional treatment (14 out of 18). One patient with an ENB refused radiotherapy and had tumor recurrence at 15 months follow-up. After a reresection and radiotherapy, the patients remain recurrence free after 51 months. In general, the prognosis is positively affected by local tumor control and the absence of distant metastasis. In this cohort, eight patients had a tumor recurrence during follow-up. Two patients received a reresection followed by adjuvant therapy, four were referred for systemic therapy, and two were given palliative care. Five of these patients died shortly after diagnosis, one is without tumor recurrence, and two showed stable disease. Kaplan et al 2 reported on prognostic factors concerning the resection of recurrent tumors. They state that histology grade, involvement of regional critical structures such as the orbit, carotid artery, and skull base negatively affect the prognosis. This study only included grade 4 tumors with skull base involvement, which could explain the severe prognosis of our recurrent tumor group.

### Complications

The treatment of high-grade, locally advanced sinonasal cancers is accompanied by high rates of complications, morbidity, and mortality. 18 Frequently reported complications are wound infections, liquorrhea, orbital deficits, radionecrosis, and systemic complications, in total up to 35%. Patients suffering from complications may no longer be eligible for adjuvant therapies, drastically diminishing their prognosis. Additionally, it negatively affects their quality of life. In our study, we observed no early wound infection, one meningitis, and five late wound infections. Three patients suffered from direct postoperative liquorrhea and one late liquorrhea, none with long-term sequela. Two patients suffered cardiovascular complications. Mortality was 3 out of 18 patients, and all complication rates are in line with reports from the literature. 18 We observed in 7 out of 14 patients complications following radiotherapy. All wound complications originated postradiotherapy, most of them needing a reoperation. Ganly et al 18 reported on these surgery-related complications and found that the addition of radiotherapy increases the rate of wound complications. Furthermore, intracranial invasion increases the risk of liquorrhea. In our cohort, all patients had intracranial expansion and were treated with adjuvant radiotherapy, which could explain the high numbers of complications.

### Limitations

The retrospective nature of this study is subject to bias, which means we need to be careful of interpreting causality. Furthermore, the high variability in tumor histology in this cohort makes it difficult to compare the clinical course and outcomes between each pathology.

## Conclusion

Patients with sinonasal cancers with skull base extension are a very rare and specific subgroup with a high recurrence rate. Due to an often late presentation, they present with advanced disease with growth into critical surrounding structures such as the orbit and brain parenchyma. This makes it more difficult to achieve a gross total resection, requiring radiation dosage to the surgical cavity resulting in a high dose to nearby healthy tissue. In this study, which includes only stage four sinonasal cancers with skull base extension, we report a survival of 50% with a mean follow-up of 49 months. This is comparable with other studies, which also include lower stages. Multimodal treatment complications are common and can be severe requiring readmission, reoperation, and rehabilitation. This study aims to add to currently available cohorts in the literature.

## References

[1] Turner J H Reh D D Incidence and survival in patients with sinonasal cancer: a historical analysis of population-based data Head Neck 2012 34 06 877 885 22127982 10.1002/hed.21830

[2] Kaplan D J Kim J H Wang E Snyderman C Prognostic indicators for salvage surgery of recurrent sinonasal malignancy Otolaryngol Head Neck Surg 2016 154 01 104 112 26424747 10.1177/0194599815606699

[3] López F Lund V J Suárez C The impact of histologic phenotype in the treatment of sinonasal cancer Adv Ther 2017 34 10 2181 2198 28871554 10.1007/s12325-017-0605-9

[4] Mendenhall W M Amdur R J Morris C G Carcinoma of the nasal cavity and paranasal sinuses Laryngoscope 2009 119 05 899 906 19358246 10.1002/lary.20196

[5] Komotar R J Starke R M Raper D MS Anand V K Schwartz T H Endoscopic endonasal compared with anterior craniofacial and combined cranionasal resection of esthesioneuroblastomas World Neurosurg 2013 80(1-2):148 159 23228365 10.1016/j.wneu.2012.12.003

[6] Hanna E DeMonte F Ibrahim S Roberts D Levine N Kupferman M Endoscopic resection of sinonasal cancers with and without craniotomy: oncologic results Arch Otolaryngol Head Neck Surg 2009 135 12 1219 1224 20026819 10.1001/archoto.2009.173

[7] Guntinas-Lichius O Kreppel M P Stuetzer H Semrau R Eckel H E Mueller R P Single modality and multimodality treatment of nasal and paranasal sinuses cancer: a single institution experience of 229 patients Eur J Surg Oncol 2007 33 02 222 228(EJSO) 17127030 10.1016/j.ejso.2006.10.033

[8] Thorup C Sebbesen L Danø H Carcinoma of the nasal cavity and paranasal sinuses in Denmark 1995-2004 Acta Oncol 2010 49 03 389 394 20001493 10.3109/02841860903428176

[9] Dirix P Nuyts S Geussens Y Malignancies of the nasal cavity and paranasal sinuses: long-term outcome with conventional or three-dimensional conformal radiotherapy Int J Radiat Oncol Biol Phys 2007 69 04 1042 1050 17570610 10.1016/j.ijrobp.2007.04.044

[10] Hoppe B S Stegman L D Zelefsky M J Treatment of nasal cavity and paranasal sinus cancer with modern radiotherapy techniques in the postoperative setting—the MSKCC experience Int J Radiat Oncol Biol Phys 2007 67 03 691 702 17161557 10.1016/j.ijrobp.2006.09.023

[11] Allen M W Schwartz D L Rana V Long-term radiotherapy outcomes for nasal cavity and septal cancers Int J Radiat Oncol Biol Phys 2008 71 02 401 406 18164845 10.1016/j.ijrobp.2007.10.031 PMC2692674

[12] Chen A M Daly M E Bucci M K Carcinomas of the paranasal sinuses and nasal cavity treated with radiotherapy at a single institution over five decades: are we making improvement? Int J Radiat Oncol Biol Phys 2007 69 01 141 147 17459609 10.1016/j.ijrobp.2007.02.031

[13] Hoppe B S Wolden S L Zelefsky M J Postoperative intensity-modulated radiation therapy for cancers of the paranasal sinuses, nasal cavity, and lacrimal glands: technique, early outcomes, and toxicity Head Neck 2008 30 07 925 932 18302261 10.1002/hed.20800

[14] Batth S S Sreeraman R Dienes E Clinical-dosimetric relationship between lacrimal gland dose and ocular toxicity after intensity-modulated radiotherapy for sinonasal tumours Br J Radiol 2013 86(1032):2.0130459E7 10.1259/bjr.20130459 PMC3856547 24167183

[15] Licitra L Resteghini C Bossi P The evolving role of systemic therapy in the primary treatment of sinonasal cancer Adv Otorhinolaryngol 2020 84 78 86 32731241 10.1159/000457927

[16] Haerle S K Gullane P J Witterick I J Zweifel C Gentili F Sinonasal carcinomas: epidemiology, pathology, and management Neurosurg Clin N Am 2013 24 01 39 49 23174356 10.1016/j.nec.2012.08.004

[17] Taylor M A Saba N F Cancer of the paranasal sinuses Hematol Oncol Clin North Am 2021 35 05 949 962 34226078 10.1016/j.hoc.2021.05.006

[18] Ganly I Patel S G Singh B Complications of craniofacial resection for malignant tumors of the skull base: report of an International Collaborative Study Head Neck 2005 27 06 445 451 15825205 10.1002/hed.20166

[19] Freites-Martinez A Santana N Arias-Santiago S Viera A Using the Common Terminology Criteria for Adverse Events (CTCAE—Version 5.0) to evaluate the severity of adverse events of anticancer therapies Actas Dermosifiliogr (Engl Ed) 2021 112 01 90 92 32891586 10.1016/j.ad.2019.05.009

[20] Mehta G U Passer J Z Raza S M The neurosurgical management of sinonasal malignancies involving the anterior skull base: a 28-year experience at The MD Anderson Cancer Center J Neurosurg 2021 136 06 1583 1591 34624857 10.3171/2021.5.JNS21772

[21] Xu C C Dziegielewski P T McGaw W T Seikaly H Sinonasal undifferentiated carcinoma (SNUC): the Alberta experience and literature review J Otolaryngol Head Neck Surg 2013 42 01 2 23663264 10.1186/1916-0216-42-2 PMC3646548

[22] Higgins T S Thorp B Rawlings B A Han J K Outcome results of endoscopic vs craniofacial resection of sinonasal malignancies: a systematic review and pooled-data analysis Int Forum Allergy Rhinol 2011 1 04 255 261 22287429 10.1002/alr.20051

[23] Lee M M Vokes E E Rosen A Witt M E Weichselbaum R R Haraf D J Multimodality therapy in advanced paranasal sinus carcinoma: superior long-term results Cancer J Sci Am 1999 5 04 219 223 10439167

[24] Orlandi E Cavalieri S Granata R Locally advanced epithelial sinonasal tumors: The impact of multimodal approach Laryngoscope 2020 130 04 857 865 31369156 10.1002/lary.28202

[25] Bossi P Saba N F Vermorken J B The role of systemic therapy in the management of sinonasal cancer: a critical review Cancer Treat Rev 2015 41 10 836 843 26255226 10.1016/j.ctrv.2015.07.004

